# Entanglement and electronic coherence in attosecond molecular photoionization

**DOI:** 10.1038/s41586-026-10230-2

**Published:** 2026-04-01

**Authors:** L.-M. Koll, A. J. Suñer-Rubio, T. Witting, R. Y. Bello, A. Palacios, F. Martín, M. J. J. Vrakking

**Affiliations:** 1 Max-Born-Institut (MBI), Berlin, Germany; 2 Departamento de Química, Módulo 13, Universidad Autónoma de Madrid, Madrid, Spain; 3 Departamento de Química Física Aplicada, Módulo 14, Universidad Autónoma de Madrid, Madrid, Spain; 4 Instituto de Física de la Materia Condensada (IFIMAC), Universidad Autónoma de Madrid, Madrid, Spain; 5 Instituto Madrileño de Estudios Avanzados en Nanociencia (IMDEA Nanociencia), Madrid, Spain

**Keywords:** Quantum mechanics, Attosecond science

## Abstract

Electronic coherences resulting from molecular photoionization underlie the process of attosecond charge migration, widely investigated as a possible path towards controlled charge-directed reactivity^1–4^. However, photoionization often creates entangled ions and photoelectrons. This entanglement compromises the ability to explore coherent ultrafast electron dynamics within ions or of their accompanying photoelectrons^5–8^. Here we present experiments and calculations in which hydrogen molecules are ionized by the combination of a phase-locked pair of isolated attosecond laser pulses and a few-cycle near-infrared (NIR) laser pulse. The electronic coherence in the dissociating H_2_^+^ ion is influenced by ion–photoelectron entanglement. We demonstrate experimental control over the degree of entanglement by varying the delay between the two attosecond pulses and the delay between these pulses and the few-cycle NIR pulse. Our work demonstrates the importance of proper consideration of the role of quantum entanglement for the optimal observation of electronic coherences in attosecond experiments.

## Abstract (web summary)

Dissociative ionization of H_2_ molecules by the combination of a phase-locked attosecond laser pair and a few-cycle NIR laser shows that ion–photoelectron entanglement influences electronic coherence in H_2_^+^, allowing control over the degree of entanglement by varying the delay between the pulses.

## Main

**Fig. 1:**
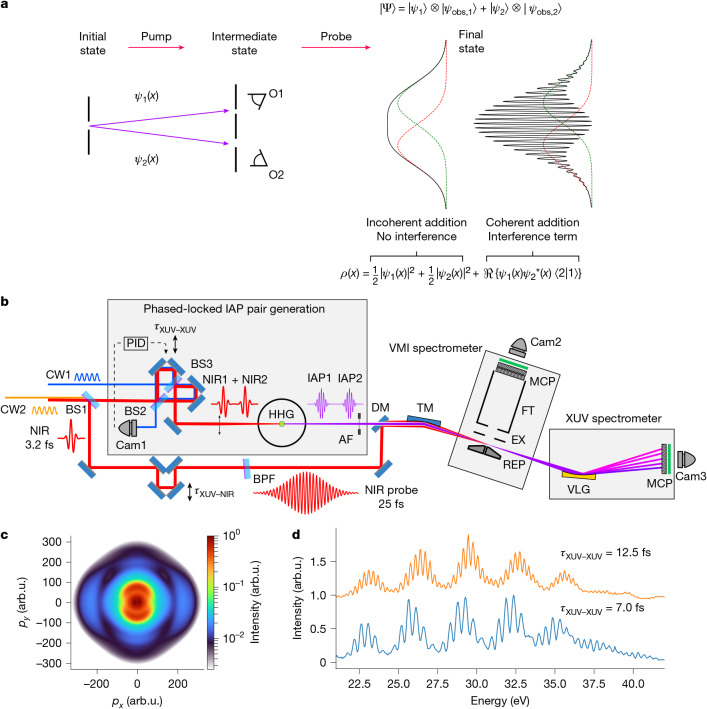
Experimental concept and approach. **a**, Time-resolved pump–probe experiments rely on interference, in which each interfering path corresponds to a coherently prepared intermediate state. Observation of the coherent evolution is possible if, and only if, the quantum path cannot be identified. In an entangled ion–photoelectron pair, a photoelectron measurement can provide information on the ionic quantum state, compromising the observation of coherent ionic dynamics. This situation resembles that of the passage of a quantum particle through a pair of slits monitored by two observers (O1 and O2): the modulation depth in the interference pattern is inversely proportional to the overlap between the observations by observers O1 and O2 (see, for example, ref. ^39^). **b**, Experimental set-up: a pair of IAPs, created by HHG, and a few-cycle NIR pulse are used to dissociatively ionize H_2_. The left–right asymmetry in the H^+^ ejection along the XUV/NIR polarization axis is measured using a VMI spectrometer and is used to quantify the electronic coherence in the dissociating H_2_^+^ ion. AF, aluminium filter; BPF, band-pass interference filter; BS, beam splitter; Cam, camera; CW, continuous-wave laser; DM, drilled mirror; EX, extractor; FT, flight tube; NIR, near-infrared laser; PID, proportional-integral-derivative controller; REP, repeller; TM, toroidal mirror; VLG, variable line-space grating. **c**, Typical VMI measurement: the 3D H^+^ momentum distribution is obtained by Abel inversion of the measured 2D projection. **d**, Typical XUV spectra recorded during the experiments, consisting of broad harmonics with a separation of about 3 eV on a continuous background, consistent with the formation of a dominant IAP with a very low intensity of the adjacent XUV pre- or post-pulses. The observed narrow fringe structure depends on the delay between the two IAPs *τ*_XUV–XUV_. arb.u., arbitrary units.

Attosecond pulses produced by high-harmonic generation (HHG) consisting of extreme-ultraviolet (XUV) radiation can ionize any conceivable compound, leading to the formation of a bipartite ion–photoelectron system that is entangled whenever the total wavefunction cannot be written as a single direct product: $|{\varPsi }_{{\rm{total}}}(t)\rangle \,\ne$ $|{\varPsi }_{{\rm{ion}}}(t)\rangle \otimes |{\phi }_{{\rm{photoelectron}}}(t)\rangle$. This occurs routinely in ionization experiments with narrowband light sources, in which the ion may be left in different eigenstates, each accompanied by photoelectrons with corresponding, well-defined kinetic energies. Ultrashort pulses excite coherent superpositions of states, creating a path towards observation of their time-resolved dynamics. This concept is taken to the extreme in attosecond science, in which bandwidths spanning several tens of eV permit the coherent excitation of several electronic configurations and the creation of electronic wave packets. Attosecond laser-induced ionization can initiate correlated dynamics of the ion and the photoelectron or in the individual subsystems. In the latter case, examining coherent dynamics in the ion (photoelectron) is only possible if a correlated observation of the accompanying photoelectron (ion) does not enable identification of the ion’s (photoelectron’s) quantum state. This situation may be compared with a multi-slit interference experiment, in which a (partial) observation of the slit through which a quantum particle moves reduces or completely removes the interference pattern on a detector: similarly, the existence of an ‘observer’ holding quantum path information compromises the coherence required for observation of a pump–probe signal (Fig. 1a). In other words, coherent dynamics in the ion or photoelectron subsystem is only possible if it is not compromised by quantum entanglement.

Building on several early results^5,6,9–11^, recent research aims to achieve a better understanding of the role of quantum entanglement^7,8,12–16^ and other sources of decoherence^17^ in attosecond experiments. This includes previous work on H_2_, investigating the relationship between ion–photoelectron entanglement and the occurrence of vibrational coherence^7,8^, as well as observations of molecular frame asymmetries in the ejection of photoelectrons^15^. In the former work, vibrational wave packets were formed in H_2_^+^ by ionizing neutral H_2_ with a pair of attosecond pulse trains and the degree of entanglement with the accompanying photoelectrons was measured by dissociating the ions, at a variable delay, using a few-cycle NIR pulse.

A main objective in attosecond molecular science is, however, the observation of ‘electronic’ coherences in ions formed by attosecond photoionization, commonly referred to as ‘attosecond charge migration’. Its interest arises from the fact that, by eliciting an electronic response on timescales preceding nuclear motion^1,18^, charge-directed reactivity^2^, that is, controlled chemistry, may be achieved. Several successful experiments have been reported^3,4,19,20^. However, the precise role of entanglement and its potential use to control coherent charge dynamics is unknown.

Ideally, studies of ion–photoelectron entanglement would use coincident detection of the ions exhibiting electronic coherence together with their corresponding photoelectrons. However, experiments combining the use of isolated attosecond pulses (IAPs) and coincident electron-ion detection have not yet been realized. Therefore, we focus on the dependence of the degree of (1) electronic coherence in an ion and (2) quantum entanglement between the ion and the photoelectron on, first, the delay between a pair of IAPs used to produce the ion and, second, the delay of a co-propagating NIR pulse. We present experiments and theoretical modelling on H_2_, showing how the kinetic energy and—in particular—the orbital angular momentum of the outgoing photoelectron, control the ion–photoelectron entanglement and electronic coherence in the ion.

**Fig. 2:**
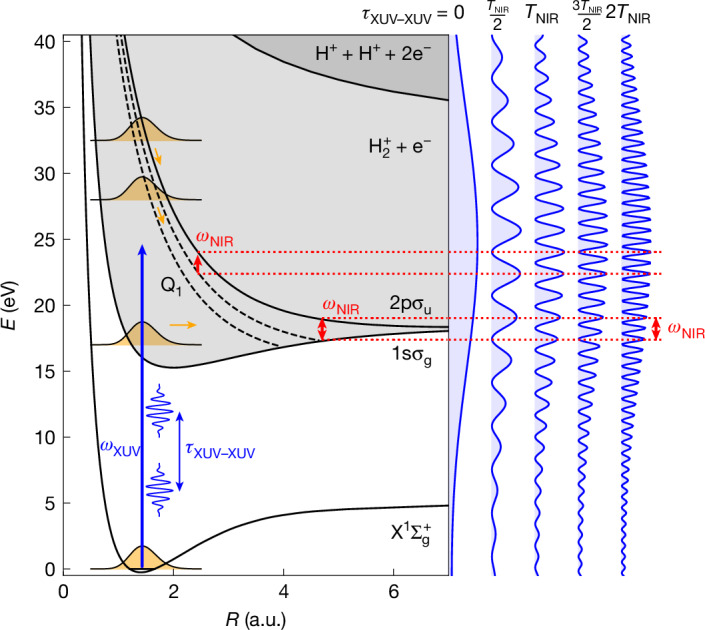
Potential curves of H_2_ and concept of the experiment. Left, relevant potential energy curves of the H_2_ molecule: the ${{\rm{X}}}^{1}\,{\sum }_{{\rm{g}}}^{+}$ ground state, the 1s*σ*_g_ and 2p*σ*_u_ ionization thresholds (that is, the ground and first excited states of the remaining molecular cation H_2_^+^), the Q_1_ and Q_2_ series of resonant autoionizing states and the double-ionization threshold. The pale grey shaded area represents the ionization continuum and the more intense grey shaded area the double-ionization continuum. A pair of identical approximately 250-attosecond-long XUV pulses with central frequency 25 eV, delayed by *τ*_XUV–XUV_, ionize the molecule from the ${{\rm{X}}}^{1}\,{\sum }_{{\rm{g}}}^{+}$ ground state (vertical blue arrow). Right, the spectra of the XUV pulse pair at selected delays are shown. For non-zero *τ*_XUV–XUV_, the XUV spectrum is modulated with a frequency $\Delta {\omega }_{{\rm{XUV}}}=\frac{2{\rm{\pi }}}{{\tau }_{{\rm{XUV}}-{\rm{XUV}}}}$. Owing to the large bandwidth of the XUV pulses, ionization leads to photoelectrons with a wide range of kinetic energies, and nuclear wave packets, represented by orange Gaussian shapes, are launched in the 1s*σ*_g_ and 2p*σ*_u_ ionization continua and the Q autoionizing states. The NIR pulse induces transitions between the ionic states and between the Q autoionizing states and the 2p*σ*_u_ continuum (small red arrows). This creates the possibility to generate a photoelectron wavefunction that is common to the 1s*σ*_g_ and 2p*σ*_u_ channels and hence a coherent superposition state of the molecular cation. The efficiency of the latter process depends on *τ*_XUV–XUV_. When *τ*_XUV–XUV_ is an integer multiple of the NIR period, that is, *ω*_*NIR*_ = *N*Δ*ω*_XUV_, pairs of XUV photons differing in energy by the energy of one NIR photon can readily be found, favouring the appearance of electronic coherence in the H_2_^+^ cation. By contrast, when *τ*_XUV–XUV_ is a half-integer multiple of the NIR period, that is, ${\omega }_{{\rm{N}}{\rm{I}}{\rm{R}}}=\left(N\pm \frac{1}{2}\right)\Delta {\omega }_{{\rm{X}}{\rm{U}}{\rm{V}}}$, this is more difficult. a.u., atomic units.

**Fig. 3:**
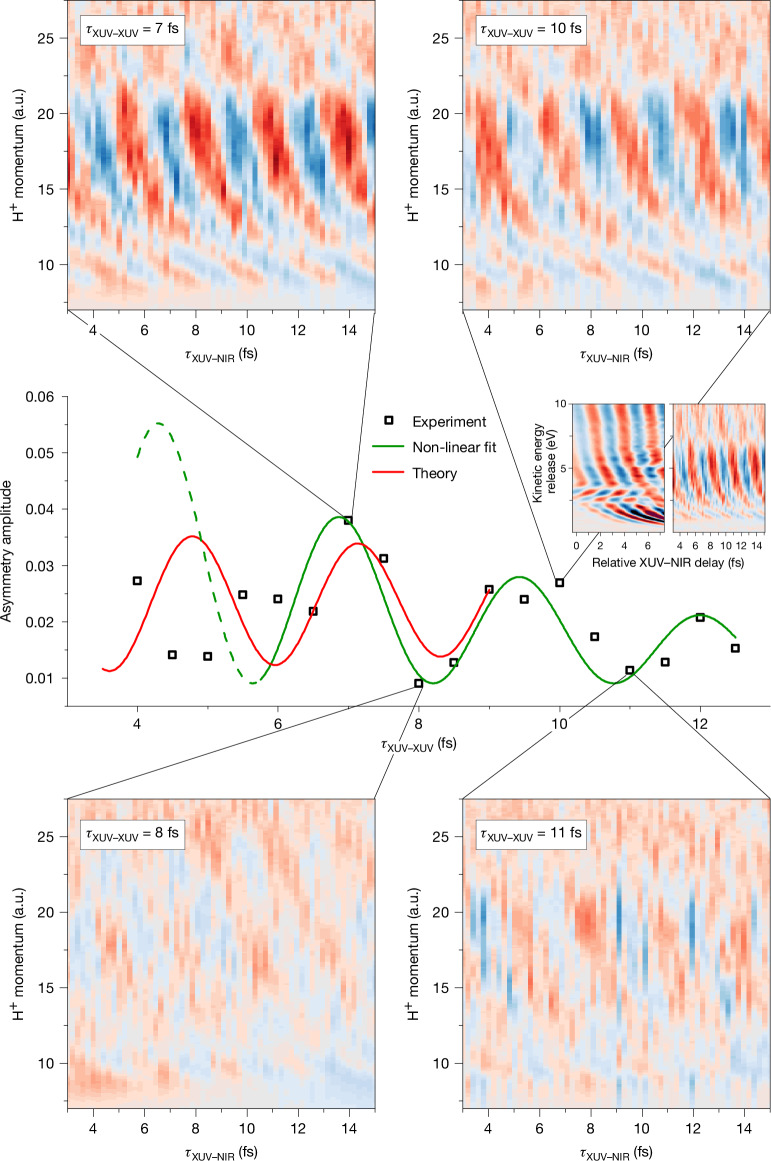
Comparison between the experimental and theoretical results. Top and bottom, normalized difference between the number of H^+^ fragment ions flying left or right along the XUV/NIR polarization axis as a function of the H^+^ momentum and the delay *τ*_XUV–NIR_ between the XUV and NIR lasers, for four different values of *τ*_XUV–XUV_, the delay between the two IAPs. The asymmetry is shown on a linear colour scale between −0.15 (dark blue) and +0.15 (dark red). The H^+^ momentum is given in atomic units. Note that uncertainties in the calibration of the VMI spectrometer introduce an uncertainty of up to 10% in the absolute values shown. For *τ*_XUV–XUV_ = 7 and 10 fs, a large amplitude of the asymmetry oscillation is observed, whereas for *τ*_XUV–XUV_ = 8 and 11 fs, the asymmetry oscillates with a greatly reduced amplitude. Middle, average amplitude of the asymmetry oscillations as a function of *τ*_XUV–XUV_. The asymmetry amplitude oscillates with a period that corresponds to the optical period of the NIR laser *T*_NIR_. The green curve results from a non-linear least squares fit and is described by *y* = 0.098 exp(−0.174*τ*_XUV–XUV_) × cos(1.223*τ*_XUV–XUV_ − 5.325)^2^ + 0.009. An oscillation frequency of 1.223 fs^−1^ corresponds to an oscillation period of 2.57 fs, close to the NIR optical period *T*_NIR_. The amplitude reduction for larger *τ*_XUV–XUV_ is because of the finite pulse duration of the NIR laser (about 25 fs). To mimic the experimental results, the theoretical results that are shown (red curve), which were obtained for a pulse duration of 15 fs, have been renormalized to the envelope of the experimental 25-fs pulse. a.u., atomic units.

Dissociative ionization by photons below about 35 eV (Fig. 2) induces fragmentation into H^+^ + H and provides a direct signature of electronic coherence in the ionic subsystem through the phenomenon of electron localization, that is, a laboratory-frame asymmetry in the ejection of the H^+^ fragment ion signifying a preferred localization of the single remaining bound electron. Following dissociation, the two lowest electronic states of H_2_^+^ can be written as

1 $${\psi }_{1{\rm{s}}{\sigma }_{{\rm{g}}}}=\frac{1}{\sqrt{2}}[{\psi }_{1{\rm{s}}}^{{\rm{left}}}+{\psi }_{1{\rm{s}}}^{{\rm{right}}}],\,{\psi }_{2{\rm{p}}{\sigma }_{{\rm{u}}}}=\frac{1}{\sqrt{2}}[{\psi }_{1{\rm{s}}}^{{\rm{left}}}-{\psi }_{1{\rm{s}}}^{{\rm{right}}}]$$

in which ${\psi }_{1{\rm{s}}}^{{\rm{left}}}$ and ${\psi }_{1{\rm{s}}}^{{\rm{right}}}$ represent 1s atomic orbitals on the left and right atoms, respectively. Rewriting this to

2 $${\psi }_{1{\rm{s}}}^{{\rm{left}}}=\frac{1}{\sqrt{2}}[{\psi }_{1{\rm{s}}{\sigma }_{{\rm{g}}}}+{\psi }_{2{\rm{p}}{\sigma }_{{\rm{u}}}}],\,{\psi }_{1{\rm{s}}}^{{\rm{right}}}=\frac{1}{\sqrt{2}}[{\psi }_{1{\rm{s}}{\sigma }_{{\rm{g}}}}-{\psi }_{2{\rm{p}}{\sigma }_{{\rm{u}}}}]$$

illustrates that asymmetries in the H^+^ ejection reflect the existence of a coherent superposition of the 1s*σ*_g_ and 2p*σ*_u_ states. Electron localization has been observed using strong-field ionization by linearly polarized^21^ and circularly polarized^22^ laser pulses and in two-colour laser fields^15,23–25^, including our earlier work combining an IAP and a few-cycle NIR field^24^, without however considering the role of entanglement. A new feature of the present experiment is its use of a phase-locked pair of IAPs. In the experiment (Methods), H_2_ was ionized by an IAP pair (*hν* ≤ 45 eV), with a variable relative delay *τ*_XUV–XUV_ ∈ ⟨4, 12.5 fs⟩. A 25-fs-long NIR pulse (about 10^12^ W cm^−2^) followed the two IAPs after a delay *τ*_XUV–NIR_ ∈ ⟨3, 15 fs⟩, with *τ*_XUV–NIR_ defined as the delay between the second IAP and the peak of the NIR pulse (with an uncertainty ±1 fs). H^+^ fragments were measured using a velocity map imaging (VMI) spectrometer^26^ and the asymmetry along the common XUV/NIR polarization axis was determined (Methods). In Fig. 3, the H^+^ fragment asymmetry is shown as a function of *τ*_XUV–NIR_ and the H^+^ momentum, for four different *τ*_XUV–XUV_. As illustrated in Fig. 2, the slowest H^+^ fragments (kinetic energy release (KER) ≤ 1 eV) are formed by XUV-only dissociative ionization on the 1s*σ*_g_ potential energy curve. Intermediate KER values are produced by resonant excitation of the neutral doubly excited Q_1_ state followed by autoionization and the highest KER values are mostly produced by dissociation on the 2p*σ*_u_ potential energy curve, which is reached by either XUV single-photon ionization or NIR ionization of the Q_1_ states. Notably, in all four cases shown, the measurement reveals an asymmetry that oscillates as a function of *τ*_XUV–NIR_ with a momentum-dependent phase, in agreement with the results reported in ref. ^24^. We note that the use of IAPs is essential for obtaining this result, because ionization by pre- and post-pulses emitted at adjacent NIR half-cycles would reduce or even cancel the observed asymmetry. In Fig. 3, the asymmetry oscillations are very pronounced for *τ*_XUV–XUV_ = 7 or 10 fs and relatively weak for *τ*_XUV–XUV_ = 8 or 11 fs. To show this more clearly, a fit of the asymmetry oscillations was performed for each H^+^ momentum. Momentum-averaged oscillation amplitudes are shown in the middle of Fig. 3, along with a fit for *τ*_XUV–XUV _≥ 6 fs. Smaller *τ*_XUV–XUV_ were rejected owing to non-negligible interference of the two NIR driver pulses during the HHG process. The average asymmetry amplitude oscillates as a function of *τ*_XUV–XUV_ with a period equal to the NIR laser optical period *T*_NIR_. The decay of the amplitude for increasing *τ*_XUV–XUV_ is caused by the finite duration of the NIR pulse. We emphasize that, in contrast with ref. ^8^, the results shown in Fig. 3 are influenced by the existence of entanglement after the combined XUV + NIR interaction (as opposed to the entanglement that is investigated in ref. ^8^ after the XUV ionization).

The results in Fig. 3 can be understood in terms of entanglement. First we note that the observation of a H^+^ fragment asymmetry requires the involvement of one or more NIR photons: after all, owing to dipole selection rules, formation of the 1s*σ*_g_ and 2p*σ*_u_ electronic states by the attosecond pulse pair is accompanied by the ejection of photoelectrons with odd and even orbital angular momentum, respectively:

3 $$\begin{array}{c}\begin{array}{c}{\varPsi }_{{{\rm{H}}}^{+}+{\rm{H}}+{{\rm{e}}}^{-}}({\rm{KER}};{\rm{EKE}},l)\,=\\ {a}_{1{\rm{s}}{\sigma }_{{\rm{g}}}}{({\rm{KER}};{\rm{EKE}},l={\rm{odd}})\psi }_{1{\rm{s}}{\sigma }_{{\rm{g}}}}{\chi }_{1{\rm{s}}{\sigma }_{{\rm{g}}}}({\rm{KER}})\otimes \phi ({\rm{EKE}},l={\rm{odd}})\,+\\ {a}_{2{\rm{p}}{\sigma }_{{\rm{u}}}}{({\rm{KER}};{\rm{EKE}},l={\rm{even}})\psi }_{2{\rm{p}}{\sigma }_{{\rm{u}}}}{\chi }_{2{\rm{p}}{\sigma }_{{\rm{u}}}}({\rm{KER}})\otimes \phi ({\rm{EKE}},l={\rm{even}})\end{array}\end{array}$$

in which ${\chi }_{1{\rm{s}}{\sigma }_{{\rm{g}}}}({\rm{KER}})$ and ${\chi }_{2{\rm{p}}{\sigma }_{{\rm{u}}}}({\rm{KER}})$ are nuclear wavefunctions leading to dissociation of H_2_^+^ along the 1s*σ*_g_ or 2p*σ*_u_ potential energy curves at a given KER and *ϕ*(EKE, *l*) is the wavefunction of a photoelectron with kinetic energy EKE and orbital angular momentum *l*. If the XUV pulses produce ionic fragments and photoelectrons with, respectively, the same KER and EKE in both H_2_^+^ electronic states, the wavefunction will be entangled because the photoelectron orbital angular momentum differs in both cases; therefore, there will not be any electronic coherence in the molecular cation.

The role of the NIR laser is (1) to change the H_2_^+^ electronic state, converting a 1s*σ*_g_ into a 2p*σ*_u_ contribution, or vice versa (Fig. 2) or (2) to change the photoelectron orbital angular momentum, in one of several ways. The NIR laser can ionize the Q_1_ states of H_2_ before they autoionize, producing a photoelectron with *l* = odd (Fig. 2) or it can interact with the outgoing photoelectron, converting a photoelectron with *l* = even into one with *l* = odd, or vice versa^24^. All of these scenarios carry the possibility to introduce terms in the wavefunction that describe the creation of electronic coherence in the cation:

4 $$\begin{array}{c}{\varPsi }_{{{\rm{H}}}^{+}+{\rm{H}}+{{\rm{e}}}^{-}}(\mathrm{KER};\mathrm{EKE},l)\,=\\ \left[\begin{array}{c}{a}_{1{\rm{s}}{\sigma }_{{\rm{g}}}}{(\mathrm{KER};\mathrm{EKE},l)\psi }_{1{\rm{s}}{\sigma }_{{\rm{g}}}}{\chi }_{1{\rm{s}}{\sigma }_{{\rm{g}}}}(\mathrm{KER})\\ +\,{a}_{2{\rm{p}}{\sigma }_{{\rm{u}}}}{(\mathrm{KER};\mathrm{EKE},l)\psi }_{2{\rm{p}}{\sigma }_{{\rm{u}}}}{\chi }_{2{\rm{p}}{\sigma }_{{\rm{u}}}}(\mathrm{KER})\end{array}\right]\otimes \phi (\mathrm{EKE},l)\end{array}$$

Notably, these scenarios involve the absorption or emission of a NIR photon, increasing or decreasing the total energy. So that the KER and EKE in both the 1s*σ*_g_ and 2p*σ*_u_ channels can be identical, the XUV photons that initiate the dissociative ionization along the 1s*σ*_g_ and 2p*σ*_u_ potential energy curves need to differ by the energy of one NIR photon *ω*_NIR_. The ease with which two such photon pathways can be found depends on *τ*_XUV–XUV_ (Fig. 2). In the frequency domain, a non-zero *τ*_XUV–XUV_ implies an XUV spectral modulation with frequency $\Delta {\omega }_{{\rm{XUV}}}=\frac{2{\rm{\pi }}}{{\tau }_{{\rm{XUV}}-{\rm{XUV}}}}$ (Figs. 1d and 2). Electronic coherence can be readily observed when *N*Δ*ω*_XUV_ = *ω*_NIR_, that is, when ${\tau }_{{\rm{X}}{\rm{U}}{\rm{V}}-{\rm{X}}{\rm{U}}{\rm{V}}}=N\frac{2{\rm{\pi }}}{{\omega }_{{\rm{N}}{\rm{I}}{\rm{R}}}}=N{T}_{{\rm{N}}{\rm{I}}{\rm{R}}}$, that is, an integer multiple of *T*_NIR_. Conversely, electronic coherence is suppressed by entanglement when ${\tau }_{{\rm{XUV}}-{\rm{XUV}}}=\left(N\pm \frac{1}{2}\right){T}_{{\rm{NIR}}}$.

In the absence of an experimental measurement of the photoelectron and the degree of ion–photoelectron entanglement, definitive conclusions about the role of entanglement require a theoretical simulation of the experiment. Therefore, the time-dependent Schrödinger equation was solved in full dimensionality (for molecules parallel to the polarization direction) by performing a close-coupling expansion of the time-dependent wavefunction in terms of a large number of H_2_ eigenstates with Σ symmetry, that is, bound states, the 1s*σ*_g_ and 2p*σ*_u_ ionization continua and doubly excited states such as the Q_1_ states that populate the 1s*σ*_g_ state by means of autoionization (Fig. 2 and Methods). The laser parameters used in the calculations were chosen to mimic the experimental scenario as closely as possible. For computational reasons, the duration of the NIR pulse was limited to 15 fs.

The reduced ionic density matrix was constructed from the computational results by tracing out the photoelectron degrees of freedom (Methods):

5 $${\rho }_{{ii}^{{\prime} }}({\rm{K}}{\rm{E}}{\rm{R}})=\sum _{l}\int \mathrm{dEKE}\,{a}_{i}({\rm{K}}{\rm{E}}{\rm{R}};\,{\rm{E}}{\rm{K}}{\rm{E}},l){a}_{{i}^{{\prime} }}^{\ast }({\rm{K}}{\rm{E}}{\rm{R}};{\rm{E}}{\rm{K}}{\rm{E}},l)$$

in which *i* and *i*′ run over 1s*σ*_g_ and 2p*σ*_u_. Singular value decomposition allows writing the reduced ionic density matrix as the sum of two density matrices, both of which are density matrices of a pure state ${\psi }_{j}={b}_{1{\rm{s}}{\sigma }_{{\rm{g}}},j}{({\rm{KER}})\psi }_{1{\rm{s}}{\sigma }_{{\rm{g}}}}{\chi }_{1{\rm{s}}{\sigma }_{{\rm{g}}}}({\rm{KER}})+{b}_{2{\rm{p}}{\sigma }_{{\rm{u}}},j}({\rm{KER}}){\psi }_{2{\rm{p}}{\sigma }_{{\rm{u}}}}{\chi }_{2{\rm{p}}{\sigma }_{{\rm{u}}}}({\rm{KER}})$ with the singular values *λ*_*j*_ defining the relative weight:

6 $$\begin{array}{c}\rho ({\rm{KER}})={\lambda }_{1}\left[\begin{array}{cc}{\rho }_{11,1}({\rm{KER}}) & {\rho }_{12,1}({\rm{KER}})\\ {\rho }_{21,1}({\rm{KER}}) & {\rho }_{22,1}({\rm{KER}})\end{array}\right]+{\lambda }_{2}\left[\begin{array}{cc}{\rho }_{11,2}({\rm{KER}}) & {\rho }_{12,2}({\rm{KER}})\\ {\rho }_{21,2}({\rm{KER}}) & {\rho }_{22,2}({\rm{KER}})\end{array}\right]\end{array}$$

Obtaining ${b}_{1{\rm{s}}{\sigma }_{{\rm{g}}},j}({\rm{KER}})$ and ${b}_{2{\rm{p}}{\sigma }_{{\rm{u}}},j}({\rm{KER}})$ from these density matrices, the asymmetry parameter is given by

7 $$A({\rm{KER}})=\frac{\sum _{j=1,2}{\lambda }_{j}{({\rm{KER}})|{b}_{1{\rm{s}}{\sigma }_{{\rm{g}}},j}({\rm{KER}})+{b}_{2{\rm{p}}{\sigma }_{{\rm{u}}},j}({\rm{KER}})|}^{2}-\sum _{j=1,2}{\lambda }_{j}{({\rm{KER}})|{b}_{1{\rm{s}}{\sigma }_{{\rm{g}}},j}({\rm{KER}})-{b}_{2{\rm{p}}{\sigma }_{{\rm{u}}},j}({\rm{KER}})|}^{2}}{\sum _{j=1,2}{\lambda }_{j}({\rm{KER}}){|{b}_{1{\rm{s}}{\sigma }_{{\rm{g}}},j}({\rm{KER}})+{b}_{2{\rm{p}}{\sigma }_{{\rm{u}}},j}({\rm{KER}})|}^{2}+\sum _{j=1,2}{\lambda }_{j}({\rm{KER}}){|{b}_{1{\rm{s}}{\sigma }_{{\rm{g}}},j}({\rm{KER}})-{b}_{2{\rm{p}}{\sigma }_{{\rm{u}}},j}({\rm{KER}})|}^{2}}$$

Following common practice in quantum statistical mechanics, we use the von Neumann entropy *S*(*ρ*(KER)) = −tr(*ρ*(KER)ln*ρ*(KER)) to assess the degree of entanglement of the ion–photoelectron system^27^: when the system is in a pure state, *λ*_2_ = 0 (*S* = 0) and the system is maximally entangled when *λ*_1_ = *λ*_2_ (*S* = ln2).

**Extended Data Fig. 1:**
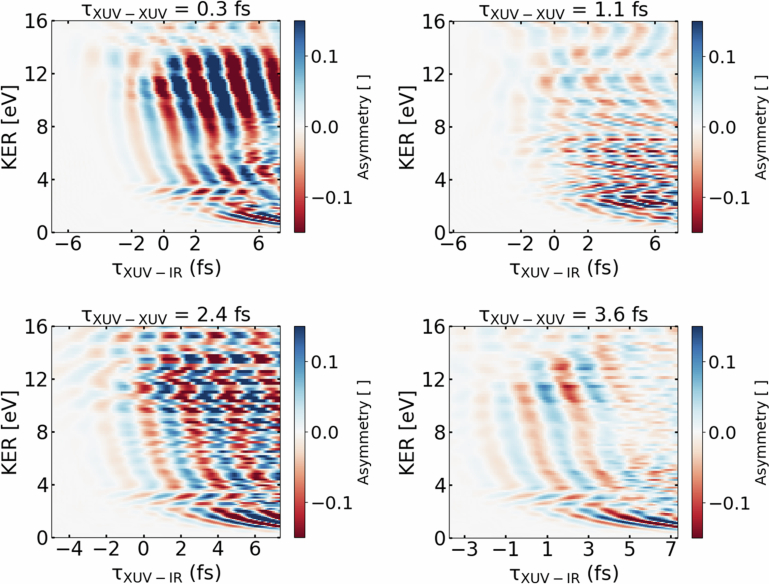
Theoretical results showing the asymmetry in the H^+^ fragment ejection as a function of the KER in the dissociation of H_2_^+^ and the relative delay between the IAP pair and the NIR laser for four different values of *τ*_XUV–XUV_.

Calculations involving a pair of IAPs were carried out as a function of *τ*_XUV–NIR_ and *τ*_XUV–XUV_ (restricted to *τ*_XUV–XUV_ ≲ 9 fs; Methods). The calculations yield the amplitudes *a*_*i*_(KER; EKE, *l*) that appear in equations (3)–(5) and are used to calculate the asymmetry and von Neumann entropy as a function of the KER using equations (6) and (7). Extended Data Fig. 1 shows the calculated H^+^ fragment asymmetry as a function of *τ*_XUV–NIR_ and the H^+^ momentum for selected *τ*_XUV–XUV_. As in the experiment, the asymmetry oscillates as a function of *τ*_XUV–NIR_ with a period *T*_NIR_ and the amplitude of the oscillations strongly depends on *τ*_XUV–XUV_. The KER-averaged asymmetry amplitude is shown as a function of *τ*_XUV–XUV_ in the middle part of Fig. 3. For *τ*_XUV–XUV_, for which both theoretical and experimental data exist, qualitatively similar behaviour is observed, namely pronounced oscillations as a function of *τ*_XUV–XUV_ with a period *T*_NIR_ and a progressive damping of these oscillations for increasing *τ*_XUV–XUV_. Quantitative differences are probably because of the use of a shorter NIR pulse in the calculations and experimental imperfections such as the existence of non-zero attosecond pre- and post-pulses.

**Fig. 4:**
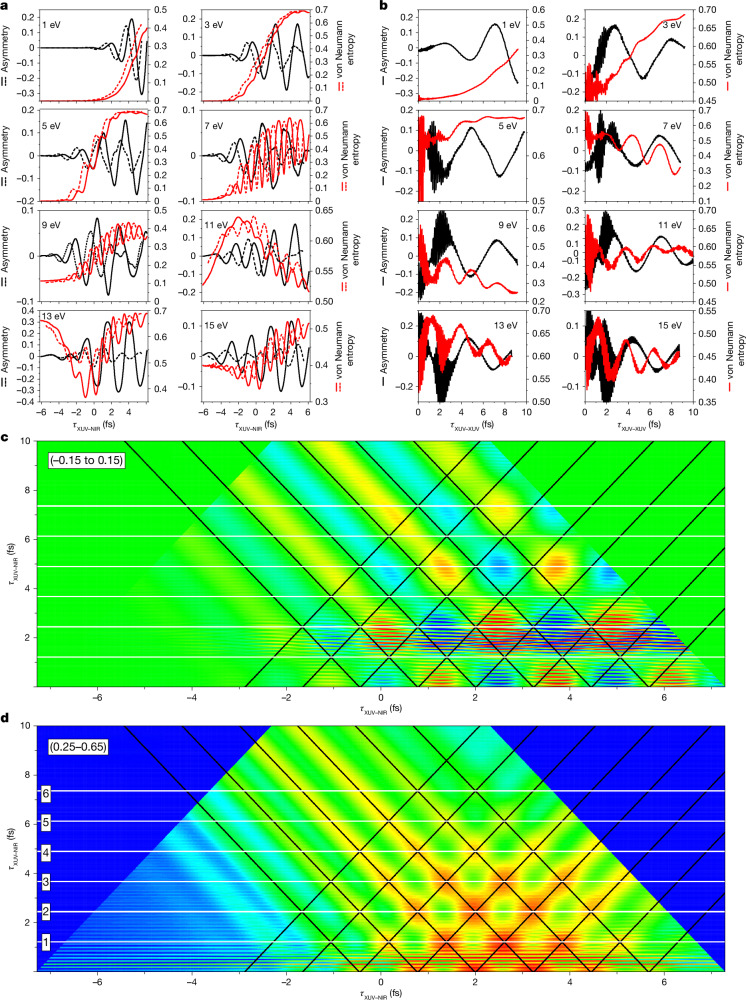
Calculations of the asymmetry and the von Neumann entropy. **a**, Asymmetry (black) and von Neumann entropy (red) as a function of the H^+^ fragment KER (indicated in each plot) and *τ*_XUV–NIR_ for two different values of *τ*_XUV–XUV_, namely *τ*_XUV–XUV_ = *T*_NIR_ (solid lines) and ${\tau }_{{\rm{XUV}}-{\rm{XUV}}}=\frac{3}{2}{T}_{{\rm{NIR}}}$ (dashed lines), in which *T*_NIR_ is the optical period of the NIR laser. **b**, Asymmetry (black) and von Neumann entropy (red) as a function of the H^+^ fragment KER (indicated in each plot) and *τ*_XUV–XUV_ for a fixed value of *τ*_XUV–NIR_, namely, *τ*_XUV–NIR_ = 2.00 fs (KER = 1 eV), 2.10 fs (KER = 3 eV), 2.40 fs (KER = 5 eV), 2.90 fs (KER = 7 eV), 2.70 fs (KER = 9 eV), 2.30 fs (KER = 11 eV), 3.00 fs (KER = 13 eV) and 2.95 fs (KER = 15 eV). **c**,**d**, Asymmetry (**c**) and von Neumann entropy (**d**) as a function of *τ*_XUV–XUV_ and *τ*_XUV–NIR_ for a H^+^ KER of 9.924 eV. The black lines that are superimposed with slopes +2 and −2 pass through the maxima in the von Neumann entropy shown in **d**; the horizontal white lines correspond to ${\tau }_{{\rm{XUV}}-{\rm{XUV}}}=M\frac{{T}_{{\rm{NIR}}}}{2}$ with *M* = 1–6. **c** and **d** are plotted on a linear colour scale over a range indicated at the top left.

In more detail, Fig. 4a,b shows, for different values of the KER, the calculated asymmetry (black curves) and von Neumann entropy (red curves) as a function of *τ*_XUV–NIR_ for *τ*_XUV–XUV_ = *T*_NIR_ (solid lines) and ${\tau }_{{\rm{XUV}}-{\rm{XUV}}}=\frac{3}{2}{T}_{{\rm{NIR}}}$ (dashed lines) and as a function of *τ*_XUV–XUV_ for selected values of *τ*_XUV–NIR_, respectively. The calculated asymmetries confirm the experimentally observed oscillatory dependencies on *τ*_XUV–XUV_ and *τ*_XUV–NIR_ and illustrate a changing role of the quantum entanglement as a function of the KER. For KER values ≤9 eV, XUV ionization predominantly produces a dissociative wave packet on the 1s*σ*_g_ potential energy curve, either by direct photoionization or by autoionization of the Q_1_ states. Without substantial population of the 2p*σ*_u_ state, the ion–photoelectron state is pure and the von Neumann entropy is zero (see Fig. 4a for *τ*_XUV–NIR_ ≪ 0 when the NIR pulse precedes the XUV pulse). For *τ*_XUV–NIR_ ≈ 0, and in particular for *τ*_XUV–NIR_ > 0, the NIR pulse populates the 2p*σ*_u_ state and a fragment asymmetry (that is, electronic coherence) is seen. In agreement with the experiment, the asymmetry in Fig. 4a oscillates with *τ*_XUV–NIR_, with two extrema (one positive, one negative) during each NIR optical period. Notably, the calculations show that the NIR also produces entanglement. This is a result of NIR interaction with the photoelectron, producing photoelectron sidebands^28^ and redistributing the orbital angular momentum over a wider range of *l*.

For KER values > 9 eV, both dissociation on the 1s*σ*_g_ potential curve (following autoionization) and direct photoionization producing the 2p*σ*_u_ state contribute. Without NIR interaction, these two quantum paths are accompanied by photoelectrons with different orbital angular momenta (equation (3)) and produce an entangled ion–photoelectron pair. Indeed, Fig. 4a now shows that, for *τ*_XUV–NIR_ ≪ 0, the von Neumann entropy is distinctly non-zero. Under the influence of the NIR, electronic coherence is created (as revealed by the asymmetry parameter) and the von Neumann entropy decreases, in particular for a KER of 13 eV. Consistent with the experiment, the asymmetry oscillations in Fig. 4a are more pronounced when *τ*_XUV–XUV_ is an integer multiple of *T*_NIR_.

In Fig. 4b, the dependence of the asymmetry and von Neumann entropy on *τ*_XUV–XUV_ are shown for selected values of *τ*_XUV–NIR_. Except for the previously discussed low KER values, the entanglement shows clear oscillatory behaviour as a function of *τ*_XUV–XUV_, with a period that is approximately equal to *T*_NIR_, in agreement with the experiment and our previous description. Very rapid oscillations with a period of about 130 attoseconds are observed, which were not seen in the experiment, which was conducted with a 200-attosecond time step. They originate from a Ramsey-type interference in the resonant excitation of the Q_1_ state.

Figure 4c,d shows the asymmetry and von Neumann entropy as a function of *τ*_XUV–XUV_ and *τ*_XUV–NIR_ for a KER of 9.924 eV. In Fig. 4d, maxima of the von Neumann entropy occur on a series of lines with slopes +2 and −2 (originating from the two pulses in the IAP pair, as revealed by calculations including only one of the two XUV pulses), with a particularly high degree of entanglement at the crossing of two such lines. By contrast, the asymmetry as a function of *τ*_XUV–XUV_ and *τ*_XUV–NIR_ (Fig. 4c) shows both positive and negative extrema for combinations of the two time delays that fall in between the black lines in which the entanglement maxima occur. The observed anticorrelation between the fragment asymmetry and the von Neumann entropy supports our interpretation that the electronic coherence is limited by ion–photoelectron entanglement and rules out interpretations of the experiment in terms of possible interference mechanisms involving only the ion.

Figure 4c,d provide further evidence for the aforementioned dependence of the electronic coherence on *τ*_XUV–XUV_. In Fig. 4d, entanglement maxima occur when ${\tau }_{{\rm{XUV}}-{\rm{XUV}}}=M\frac{{T}_{{\rm{NIR}}}}{2}$ (see white lines, labelled by *M*). For odd *M*, the electronic coherence has a minimum for all values of *τ*_XUV–NIR_, in agreement with the discussion of Fig. 3. For even *M*, the entanglement owing to the XUV spectral modulation is suppressed and maxima of the electronic coherence occur for selected values of *τ*_XUV–NIR_, in which the entanglement shows a minimum (and vice versa). We note that, although in our paper we have chosen to use a frequency-domain description, parts of our observations might also be understood using a time-domain description that considers how electronic coherences in the H_2_^+^ ion produced by the two attosecond pulses add constructively or destructively. However, such a time-domain description does not provide insight into the clear anticorrelation between electronic coherence and entanglement that we see in Fig. 4c,d.

The prominent role of quantum entanglement demonstrated in this work is probably of widespread importance in the investigation of systems with a high degree of symmetry. Moreover, whereas here and previously^7,8^ we have investigated entanglement between the photoelectron and the electronic and vibrational degrees of freedom of an ion, entanglement involving rotational degrees of freedom is expected to be important as well^29^ and is a topic of future research. Our work fits in a recent development in which the attosecond community is discovering itself as a fertile playground for the investigation of fundamental quantum mechanical and quantum optical concepts^30^, with recent work on the use of HHG for producing high-photon-number entangled states^31,32^ and on strong field processes driven using non-classical light^33,34^. Also, a new protocol for the implementation of a Bell test using ultrafast lasers has been proposed^35^. Our work may stimulate more detailed studies of the role of quantum entanglement in time-resolved spectroscopy, including studies of how entanglement can be actively controlled. Moreover, the use of a phase-locked pair of IAPs may stimulate the development of XUV multidimensional spectroscopy on attosecond timescales, extending highly fruitful use of multidimensional measurement techniques in other frequency domains^36–38^.

## Methods

### Experimental methods

In the experiment, NIR laser pulses from a carrier-envelope phase-stabilized 1-kHz Ti:Sa laser (25 fs pulse duration, 2 mJ per pulse, carrier-envelope phase stability 581 mrad r.m.s., as measured at 1 kHz with a home-built f-to-2f interferometer) were compressed to a pulse duration of 3.2 fs, as measured using the SEA-SPIDER method^40^. A 90%/10% beam splitter was used to divide the compressed NIR pulses into two parts, with the higher energy beam (‘beam A’) used for the generation of isolated attosecond laser pulses and the weaker one (‘beam B’) used in the two-colour XUV + NIR experiment. Before the HHG process, beam A was passed through a passively and actively stabilized Mach–Zehnder interferometer, in which the NIR pulse was split into a collinear, phase-locked pulse pair with a precisely controllable delay (Δ*t*_jitter,XUV–XUV_ = 8 attosecond r.m.s, as measured at 1.5 kHz sampling rate using a 473-nm continuous-wave laser)^41^. Use of these phase-locked NIR pulses in HHG led to the production of a phase-locked pair of IAPs. To limit interference between the two NIR pulses in the HHG process, NIR–NIR delays between 4 and 12.5 fs were used in the experiments. An aluminium filter was used to remove beam A after the HHG. Beam B was passed through a 40-nm band-pass filter to generate a bandwidth-limited 25-fs probe pulse and was collinearly recombined with the IAP pair using a cored mirror, with the divergence of beam B matching that of the IAP pair. The time delay between the second of the two isolated attosecond laser pulses and the NIR laser pulse was varied between 3 and 15 fs in 200-attosecond steps and was actively stabilized (Δ*t*_jitter,XUV–NIR_ < 50 attoseconds). The IAP pair and NIR beam B were refocused using a 40-cm FL grazing incidence toroidal mirror, imaging the IAP pair and beam B onto the interaction region of a VMI spectrometer^26^, in which neutral H_2_ gas was effusively injected using a 10-micron nozzle integrated into the repeller electrode^42^. The polarization of the IAP pair and the NIR pulse were parallel to each other and in the plane of the detector. H^+^ ions resulting from XUV-only and XUV + NIR dissociative ionization were imaged onto a microchannel plate (MCP) + phosphor screen assembly and subsequently recorded using a charge-coupled device camera. Recorded spatial distributions were processed by inverse Abel transformation to retrieve 3D H^+^ momentum distributions and used to construct the momentum maps and asymmetry plots shown in Figs. 1 and 3.

### Extraction of the 3D velocity distribution and the laboratory-frame asymmetry

In the VMI spectrometer, a 2D projection of the 3D velocity distribution of the ejected H^+^ fragments is measured. This 3D velocity distribution is obtained from the 2D projection by means of an inverse Abel transformation using a home-built code, which describes both the measured 2D projection and the desired 3D velocity distribution as the product of a radial velocity distribution and an angular distribution that is expressed by a superposition of Legendre polynomials:

8 $${P}_{3{\rm{D}}}({v}_{3{\rm{D}}},{\theta }_{3{\rm{D}}},{\varphi }_{3{\rm{D}}})=\sum _{l}{a}_{{v}_{3{\rm{D}},l}}{P}_{l}(\cos {\theta }_{3{\rm{D}}})$$

$${P}_{2{\rm{D}}}({v}_{2{\rm{D}}},{\theta }_{2{\rm{D}}})=\sum _{l}{b}_{{v}_{2{\rm{D}},l}}{P}_{l}(\cos {\theta }_{2{\rm{D}}})$$

In these expressions, *v*_3D_ is the particle velocity and *θ*_3D_ and *φ*_3D_ are angles describing the direction of the velocity of the particle with respect to the polarization axis of the XUV pulse pair and the NIR pulse, *v*_2D_ is the measured velocity in the plane of the MCP detector and *θ*_2D_ is the angle of this velocity with respect to the XUV/NIR polarization axis.

The asymmetry is evaluated along the polarization axis, where the count rates are very low. As a result, the 3D velocity distribution is not a smooth function of velocity but is affected by counting statistics. To overcome this, as a first step in the data analysis, Savitzky–Golay filtering (filter order = 2; filter length = 30) was applied to the experimental data. Next, apparent asymmetries arising as a result of the non-uniformity of the MCP + phosphor screen detector were addressed. Before conducting the inverse Abel transform, the raw data were corrected for each XUV–XUV time delay *τ*_XUV–XUV_ by converting the measured intensity distribution *I*(*x*, *y*), in which the *y*-direction corresponds to the direction of the XUV/NIR polarization axis, in the following manner

10 $$I(x,y)\to I(x,y)+\frac{1}{2N}\mathop{\sum }\limits_{i=1}^{N}\{I(x,-y)-I(x,y)\}$$

11 $$I(x,-y)\to I(x,-y)-\frac{1}{2N}\mathop{\sum }\limits_{i=1}^{N}\{I(x,-y)-I(x,y)\}$$

in which *N* is the number of different XUV–NIR time delays *τ*_XUV–NIR_ for which measurements were performed. This transformation guarantees that, for a given *τ*_XUV–XUV_, the intensity at position (*x*, *y*) is on average equal to that at position (*x*, −*y*).

After the Abel inversion, the experimental asymmetry plotted in Fig. 3 was determined as $A(y\approx {v}_{3{\rm{D}}})=\frac{{P}_{3{\rm{D}}}({v}_{3{\rm{D}}},{\theta }_{3{\rm{D}}}=0,{\varphi }_{3{\rm{D}}})-{P}_{3{\rm{D}}}({v}_{3{\rm{D}}},{\theta }_{3{\rm{D}}}={\rm{\pi }},{\varphi }_{3{\rm{D}}})}{{P}_{3{\rm{D}}}({v}_{3{\rm{D}}},{\theta }_{3{\rm{D}}}=0,{\varphi }_{3{\rm{D}}})+{P}_{3{\rm{D}}}({v}_{3{\rm{D}}},{\theta }_{3{\rm{D}}}={\rm{\pi }},{\varphi }_{3{\rm{D}}})}$. This asymmetry depends on *τ*_XUV–XUV_, *τ*_XUV–NIR_ and the H^+^ momentum. The dependence on *τ*_XUV–NIR_ and the H^+^ momentum is shown in Fig. 3 for selected values of *τ*_XUV–XUV_. To evaluate the dependence of the asymmetry on *τ*_XUV–XUV_, it is helpful to define a single parameter that serves as a measure of the amplitude of the asymmetry oscillations for a given *τ*_XUV–XUV_. This was done in the following way: for each value of *τ*_XUV–XUV_, first, the asymmetry as a function of *τ*_XUV–NIR_ was fitted to an oscillatory function *A* = *P*(1)cos(*ωτ*_XUV–NIR_ + *P*(2)) for each value of the H^+^ momentum; next, the asymmetry amplitudes *P*(1) in this expression were averaged over the H^+^ momentum (within the range shown in Fig. 3). The average asymmetry amplitude thus obtained is shown in the middle part of Fig. 3. The frequency *ω* in the oscillatory function was initially included in the fitting as a third fit parameter but was subsequently fixed at the average value found within these fits, namely 2.31 fs^−1^. This frequency agrees well with the frequency of the NIR laser and is consistent with the fact that the oscillations in the asymmetry are induced by the NIR field.

**Extended Data Fig. 2:**
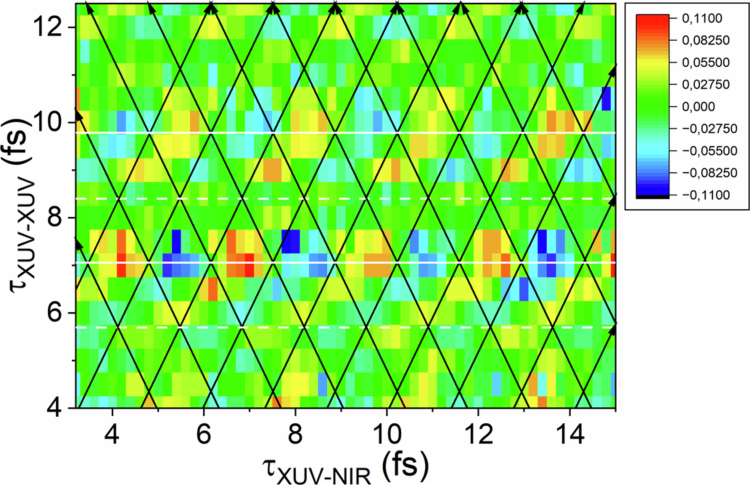
Experimentally measured asymmetry at pixel radius 200 (corresponding to a H^+^ momentum of 16.8 a.u.) as a function of *τ*_XUV–XUV_ and *τ*_XUV–NIR_. Similar to Fig. 4c, maxima in the experimental asymmetry lie in between lines with slopes +2 and −2. Also, the maxima of the asymmetry occur for specific values *τ*_XUV–XUV,max_ (solid white lines) and asymmetries are absent for values *τ*_XUV–XUV,min_ that lie halfway between two different *τ*_XUV–XUV,max_ (dashed white lines).

As well as the material that is included in Fig. 3, our experimental dataset allows us to plot the dependence of the asymmetry on *τ*_XUV–XUV_ and *τ*_XUV–NIR_ for selected values of values of the H^+^ momentum, similar to the way in which this is done in Fig. 4c for the theoretical data. For example, for the case of H^+^ momentum *k* = 16.8 a.u. (corresponding to pixel radius 200 on our camera system), this gives Extended Data Fig. 2.

**Extended Data Fig. 3:**
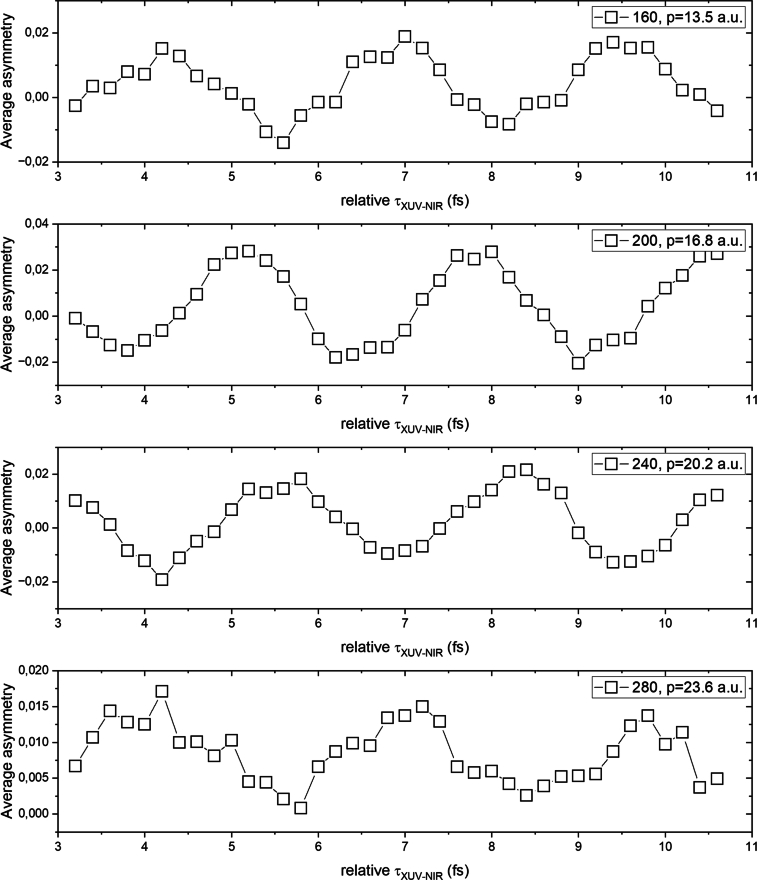
Oscillations in the measured asymmetry as a function of *τ*_XUV–NIR_, obtained by integrating the measured asymmetry as a function of *τ*_XUV–XUV_ and *τ*_XUV–NIR_ along lines with a slope of +2 (Extended Data Fig. 2). In agreement with the raw data that are shown for delays *τ*_XUV–XUV_ = 7, 8, 10 and 11 fs in Fig. 3, the asymmetry oscillates a function of *τ*_XUV–NIR_ with a period that is equal to the NIR optical period and with an amplitude that depends on the H^+^ momentum.

To further illustrate the dependence of the asymmetry on *τ*_XUV–NIR_, we have integrated these plots along lines with slope +2 (corresponding to integration parallel to the positively sloped black lines that are shown in Extended Data Fig. 2). Performing this procedure for different H^+^ momenta gives Extended Data Fig. 3, clearly showing that the H^+^ fragment asymmetry oscillates with the NIR optical period.

### Theoretical method

Our theoretical method (used in Figs. 3 and 4) closely follows that described in refs. ^43,44^. In brief, the time-dependent Schrödinger equation was solved in full dimensionality by performing a close-coupling expansion of the time-dependent wavefunction in terms of products of electronic and vibrational states of the H_2_ molecule. The light–molecule interaction was described within the dipole approximation in the length gauge. The molecule was assumed to be parallel to the polarization direction of the light pulses, so that, within the latter approximation, only electronic states of ^1^Σ^+^_g_ and ^1^Σ^+^_u_ symmetries must be considered. For each symmetry, the close-coupling expansion included the lowest six bound states of H_2_, the continuum states associated with the ^2^Σ^+^_g_ (1s*σ*_g_) and ^2^Σ^+^_u_ (2p*σ*_u_) ionization continua and the Q_1_ and Q_2_ doubly excited states embedded in these ionization continua. The electronic bound states were obtained by diagonalizing the electronic Hamiltonian in a basis of antisymmetrized products of H_2_^+^ orbitals expressed as linear combinations of products of B-splines (radial part) and spherical harmonics (angular part). The continuum states, with the proper incoming boundary conditions, were obtained by solving the scattering equations in a basis of antisymmetrized products of bound H_2_^+^ orbitals and continuum orbitals, both expressed in terms of B-splines and spherical harmonics. Finally, the vibrational wavefunctions were obtained by diagonalizing the nuclear Hamiltonians in a basis of B-splines. For the electronic part, we used a basis of 450 B-splines in a radial box of 300 a.u. and spherical harmonics with *l* ≤ 16 for the bound orbitals and *l* ≤ 11 for the continuum orbitals. The TDSE expansion included partial waves up to *l* = 7. For the vibrational and dissociative parts, a basis of 240 B-splines within a box of 12 a.u. was used. The choice of the electronic box ensures that, for *τ*_XUV–XUV_ ≤ 9 fs, no notable artificial reflection of the ejected electron in the box boundaries occurs before dissociative ionization is completed in the investigated range of *τ*_XUV–NIR_ delays.

**Extended Data Fig. 4:**
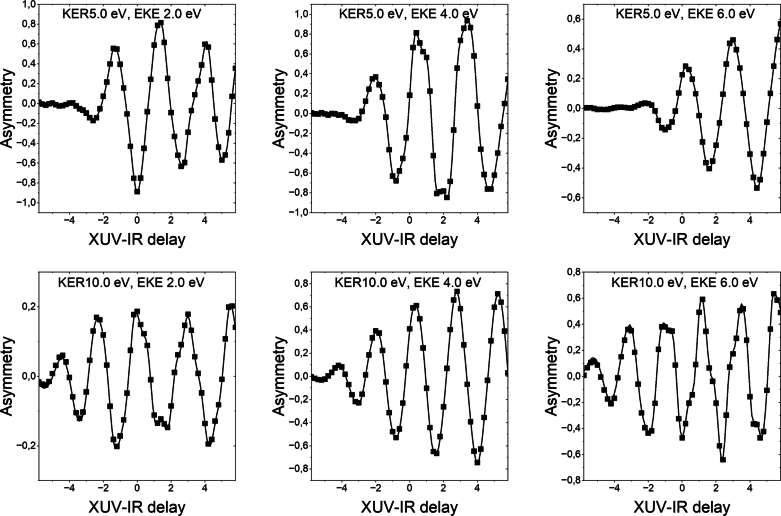
Comparison between asymmetry parameters from two-IAP calculations (squares) with *τ*_XUV–XUV_ =3 fs and asymmetry parameters calculated using amplitudes from one-IAP calculations (lines).

For a realistic comparison with the experiment, an XUV pulse with central energy of 25 eV, a duration of 250 attoseconds and a cosine-square envelope was used, along with a NIR pulse with central energy 1.65 eV (751 nm), a duration of 15 fs and a cosine-square envelope. The NIR pulse was phase-locked with the pump pulse. The peak intensities of the XUV and NIR pulses were 3 × 10^10^ W cm^−2^ and 10^11^ W cm^−2^, respectively, similar to the experimental values. A combined scan of XUV–NIR and XUV–XUV delays was performed in the intervals from −7.3 to 7.3 fs and from 0 to 10 fs, respectively, with step sizes of 0.025 fs and 0.050 fs. Negative XUV–NIR delays indicate that the NIR probe pulse precedes the XUV pump pulse. Results for XUV pulse pairs were obtained by calculating the coherent sum of transition amplitudes obtained for two different delays of a single XUV pulse with respect to the NIR pulse. This procedure is fully justified owing to the perturbative nature of the interaction between the XUV pulses and the molecule for the chosen pulse parameters. As shown in Extended Data Fig. 4, in which these asymmetry parameters are compared with results from a true two-XUV pump/NIR probe calculation, the errors associated with this procedure are negligible.

### Reduced ionic density matrix

It is convenient to analyse the results of the close-coupling calculations by constructing the reduced ionic density matrix, which, for a given KER, is given by

12 $${\rho }_{{ii}^{{\prime} }}({\rm{K}}{\rm{E}}{\rm{R}})=\sum _{l}\int \mathrm{dEKE}\,{a}_{i}({\rm{K}}{\rm{E}}{\rm{R}};{\rm{E}}{\rm{K}}{\rm{E}},l){a}_{{i}^{{\prime} }}^{\ast }({\rm{K}}{\rm{E}}{\rm{R}};{\rm{E}}{\rm{K}}{\rm{E}},l)$$

in which the labels *i* and *i*′ run over 1s*σ*_g_ and 2p*σ*_u_, and *l* and EKE are the orbital angular momentum and the kinetic energy of the photoelectron. When this density matrix is subjected to a diagonalization, here written in the general form of a singular value decomposition (necessary when applied to the wavefunction), then—afterwards—it can be written as

13 $$\begin{array}{c}\begin{array}{c}{\rho }_{{ii}{\rm{{\prime} }}}({\rm{KER}})=\sum _{j=1,2}{U}_{{ij}}{\lambda }_{j}{V}_{i{\rm{{\prime} }}j}^{\ast }\\ =\,{\lambda }_{1}({\rm{KER}})\left[\begin{array}{cc}{\rho }_{11,1}({\rm{KER}}) & {\rho }_{12,1}({\rm{KER}})\\ {\rho }_{21,1}({\rm{KER}}) & {\rho }_{22,1}({\rm{KER}})\end{array}\right]\\ \,+{\lambda }_{2}({\rm{KER}})\left[\begin{array}{cc}{\rho }_{11,2}({\rm{KER}}) & {\rho }_{12,2}({\rm{KER}})\\ {\rho }_{21,2}({\rm{KER}}) & {\rho }_{22,2}({\rm{KER}})\end{array}\right]\end{array}\end{array}$$

Each of the density matrices $$\left[\begin{array}{cc}{\rho }_{11,1}({\rm{KER}}) & {\rho }_{12,1}({\rm{KER}})\\ {\rho }_{21,1}({\rm{KER}}) & {\rho }_{22,1}({\rm{KER}})\end{array}\right]$$ and $$\left[\begin{array}{cc}{\rho }_{11,2}({\rm{KER}}) & {\rho }_{12,2}({\rm{KER}})\\ {\rho }_{21,2}({\rm{KER}}) & {\rho }_{22,2}({\rm{KER}})\end{array}\right]$$ is itself the density matrix of a pure state ${\psi }_{j}={b}_{1{\rm{s}}{\sigma }_{{\rm{g}}},j}({\rm{KER}}){\psi }_{1{\rm{s}}{\sigma }_{{\rm{g}}}}({\rm{KER}})+{b}_{2{\rm{p}}{\sigma }_{{\rm{u}}},j}{({\rm{KER}})\psi }_{2{\rm{p}}{\sigma }_{{\rm{u}}}}({KER})$, that is, must be of the form $$\left[\begin{array}{cc}{|{b}_{1{\rm{s}}{\sigma }_{{\rm{g}}},j}|}^{2} & {b}_{1{\rm{s}}{\sigma }_{{\rm{g}}},j}{b}_{2{\rm{p}}{\sigma }_{{\rm{u}}},j}^{\ast }\\ {b}_{2{\rm{p}}{\sigma }_{{\rm{u}}},j}{b}_{1{\rm{s}}{\sigma }_{{\rm{g}}},j}^{\ast } & {|{b}_{2{\rm{p}}{\sigma }_{{\rm{u}}},j}|}^{2}\end{array}\right].$$ For simplicity in the notation, in the previous expression of *Ψ*_*j*_, we have integrated in ${\psi }_{1{\rm{s}}{\sigma }_{{\rm{g}}}}$ and ${\psi }_{2{\rm{p}}{\sigma }_{{\rm{u}}}}$ the respective nuclear wavefunctions ${\chi }_{1{\rm{s}}{\sigma }_{{\rm{g}}}}$ and ${\chi }_{2{\rm{p}}{\sigma }_{{\rm{u}}}}$. It is straightforward to obtain the values of ${b}_{1{\rm{s}}{\sigma }_{{\rm{g}}},j}({\rm{KER}})$ and ${b}_{2{\rm{p}}{\sigma }_{{\rm{u}}},j}({\rm{KER}})$ (up to an arbitrary phase and normalization constant) from each of the two density matrices using

14 $$\begin{array}{c}\begin{array}{c}{b}_{1{\rm{s}}{\sigma }_{{\rm{g}}},j}({\rm{KER}})\dot{=}{({U}_{1j}{({\rm{KER}})V}_{1j}^{\ast }({\rm{KER}}))}^{\frac{1}{2}}\\ {b}_{2{\rm{p}}{\sigma }_{{\rm{u}}},j}({\rm{KER}})={b}_{1{\rm{s}}{\sigma }_{{\rm{g}}},j}({\rm{KER}})\times \frac{{U}_{2j}({\rm{KER}})}{{U}_{1j}({\rm{KER}})}\end{array}\end{array}$$

Rewriting the pure states corresponding to the two density matrices

15 $$\begin{array}{c}\begin{array}{c}{\psi }_{j}({\rm{KER}})={b}_{1{\rm{s}}{\sigma }_{{\rm{g}}},j}({\rm{KER}}){\psi }_{1{\rm{s}}{\sigma }_{{\rm{g}}}}({\rm{KER}})+{b}_{2{\rm{p}}{\sigma }_{{\rm{u}}},j}{({\rm{KER}})\psi }_{2{\rm{p}}{\sigma }_{u}}({\rm{KER}})\\ \,=\,\frac{1}{\sqrt{2}}({b}_{1{\rm{s}}{\sigma }_{{\rm{g}}},j}({\rm{KER}})+{b}_{2{\rm{p}}{\sigma }_{{\rm{u}}},j}({\rm{KER}})){\psi }_{{\rm{left}}}({\rm{KER}})+\frac{1}{\sqrt{2}}({b}_{1{\rm{s}}{\sigma }_{{\rm{g}}},j}({\rm{KER}})-{b}_{2{\rm{p}}{\sigma }_{{\rm{u}}},j}({\rm{KER}})){\psi }_{{\rm{right}}}({\rm{KER}})\end{array}\end{array}$$

leads to

16 $${P}_{{\rm{left}}}({\rm{KER}})\approx \sum _{j=1,2}{\lambda }_{j}{|{b}_{1{\rm{s}}{\sigma }_{{\rm{g}}},j}({\rm{KER}})+{b}_{2{\rm{p}}{\sigma }_{{\rm{u}}},j}({\rm{KER}})|}^{2}$$

17 $${P}_{{\rm{right}}}({\rm{KER}})\approx \sum _{j=1,2}{\lambda }_{j}{|{b}_{1{\rm{s}}{\sigma }_{{\rm{g}}},j}({\rm{KER}})-{b}_{2{\rm{p}}{\sigma }_{{\rm{u}}},j}({\rm{KER}})|}^{2}$$

resulting in an asymmetry given by the normalized difference between these two quantities (equation (7)). We would like to point out that fully equivalent results are obtained by projecting the wavefunction that results from the time-dependent Schrödinger equation onto electronic states in one of the H atoms times the nuclear state associated with the given KER^45^. We use the von Neumann entropy *S*(*ρ*(KER)) = −tr(*ρ*(KER)ln*ρ*(KER)) as a measure of the degree of entanglement; when *S* = 0, there is no entanglement between the ion and the photoelectron, whereas the ion and the photoelectron are maximally entangled when *S* = ln2.

**Extended Data Fig. 5:**
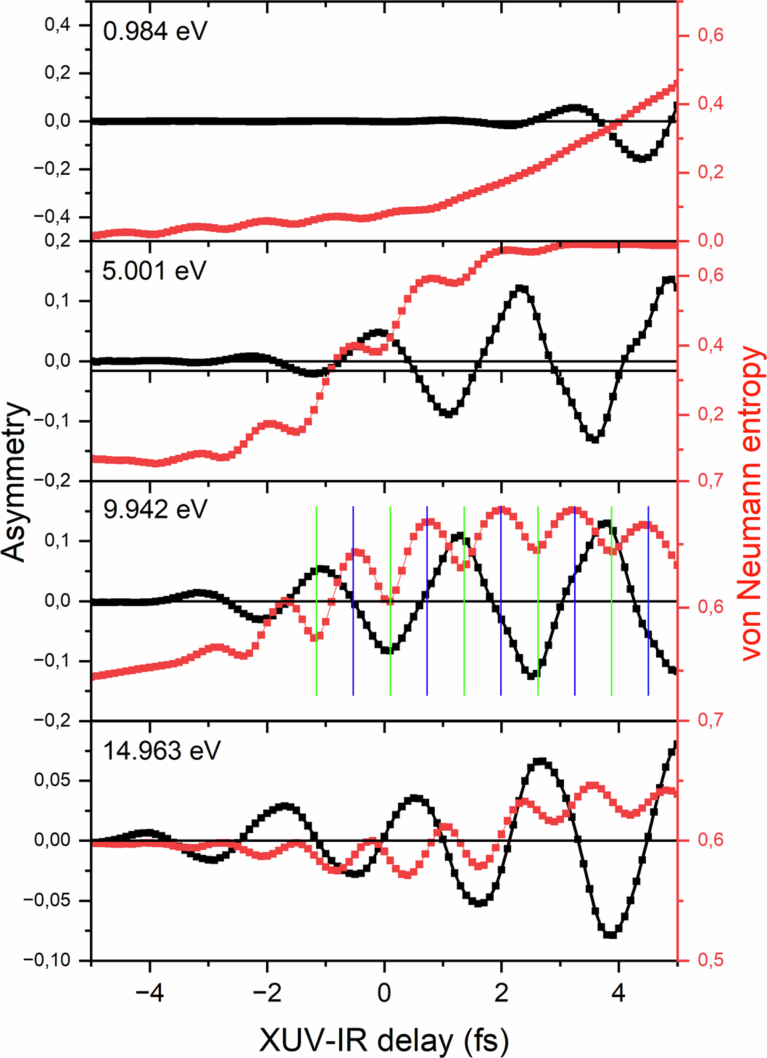
Comparison between the asymmetry parameter and the von Neumann entropy for four different values of the KER for *τ*_XUV–XUV_ = 0. For a KER of 0.984 and 5.001 eV, the asymmetry predominantly arises as a result of the formation of a coherent superposition of the 1s*σ*_g_ and 2p*σ*_u_ states by NIR absorption of a dissociative wave packet that is formed by direct photoionization to the 1s*σ*_g_ state or by autoionization of a dissociative wave packet that is launched on the doubly excited Q_1_ state. The photoelectron is not involved in this mechanism for the production of electronic coherence and ion–photoelectron entanglement is produced as a result of the formation of photoelectron sidebands. By contrast, for KERs of 9.942 and 14.963 eV, the ion–photoelectron wavefunction that is produced by ionization by the attosecond pulse is already entangled, because photoionization produces the 1s*σ*_g_ state in combination with odd and the 2p*σ*_u_ state in combination with even photoelectron orbital angular momentum. As a result of the interaction of the ion and/or photoelectron with the NIR field, electronic coherence (that is, a H^+^ fragment asymmetry) is produced, in which, in particular in the case of the 9.942 eV results, the maxima (minima) in the absolute value of the asymmetry correlate with minima (maxima) of the ion–photoelectron entanglement: at XUV–NIR delays at which the von Neumann entropy is at a maximum (blue lines), the asymmetry is close to zero, whereas minima in the von Neumann entropy (green lines) are accompanied by a maximum in the amplitude of the asymmetry, with either a positive or a negative sign.

**Extended Data Fig. 6:**
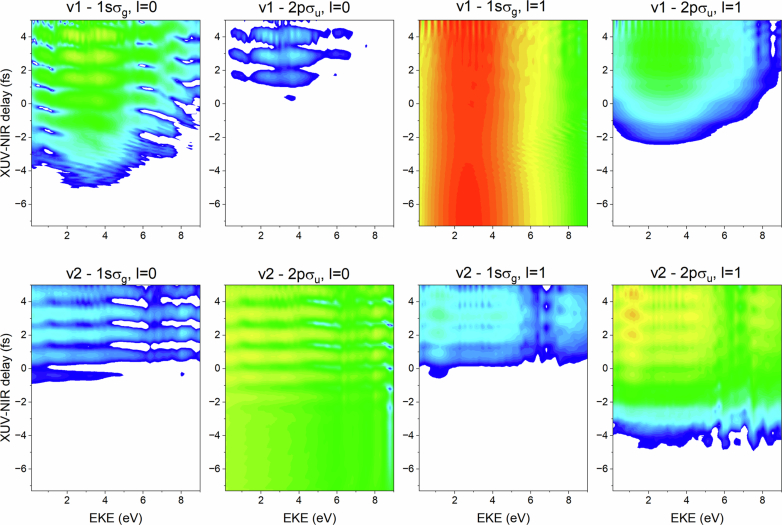
Norm of the photoelectron wavefunctions accompanying the nuclear wavefunctions associated with the first (top row, *λ*_1_) and second (bottom row, *λ*_2_) singular value in a Schmidt decomposition of the wavefunction, for a KER of 9.942 eV and *τ*_XUV–XUV_ = 0. For negative XUV–NIR time delays, when the NIR pulse precedes the XUV pulse, the ion–photoelectron wavefunction is already entangled, that is, the contributions associated with the second singular value are non-zero, owing to the fact that the 1s*σ*_g_ state is formed in combination with odd (predominantly *l* = 1, third column) and the 2p*σ*_u_ state is formed in combination with even (predominantly *l* = 0, second column) photoelectron orbital angular momentum. When the XUV and NIR pulses overlap and the delay between them is positive, electronic coherence is formed, that is, a coherent superposition of the 1s*σ*_g_ and 2p*σ*_u_ states is formed when these states are populated in combination photoelectrons that have identical properties (EKE and orbital angular momentum *l*). The observed asymmetry is mostly because of the formation of a coherent superposition of the 1s*σ*_g_ and 2p*σ*_u_ states in combination with photoelectrons with *l* = 1 (top row, third and fourth columns). The colour scale covers four orders of magnitude of intensity.

The conclusion that the wavefunction is entangled can also be obtained by performing a singular value decomposition (that is, Schmidt decomposition) of the wavefunction for a selected value of the KER. This leads to a description of the wavefunction in terms of a sum of two direct products, with singular values *μ*_1_(KER) and *μ*_2_(KER) that are related to the eigenvalues *λ*_1_(KER) and *λ*_2_(KER) in equation (13) by ${\mu }_{1}({\rm{KER}})=\sqrt{{\lambda }_{1}({\rm{KER}})}$ and ${\mu }_{2}({\rm{KER}})=\sqrt{{\lambda }_{2}({\rm{KER}})}$. The ionic and electronic eigenvectors obtained in a Schmidt decomposition of the wavefunction are the same as those obtained from singular value decomposition of the reduced ionic and electronic density matrices. A calculation of the asymmetry and von Neumann entropy for four selected values of the KER is shown in Extended Data Fig. 5. The situation for a KER of 9.942 eV is shown in Extended Data Fig. 6.

## Online content

Any methods, additional references, Nature Portfolio reporting summaries, source data, extended data, supplementary information, acknowledgements, peer review information; details of author contributions and competing interests; and statements of data and code availability are available at 10.1038/s41586-026-10230-2.

## Data Availability

The experimental and theoretical data used to prepare Fig. 3 and the numerical data used to prepare Fig. 4 can be accessed at 10.5281/zenodo.18472517 (ref. ^46^).

## Code availability

The set of codes used for the theoretical calculations and simulations (see Figs. 3 and 4) requires access to tens of millions of CPU hours in high-performance computers (such as the one acknowledged here) and cannot be run without specialized training by the developers. The authors will be happy to enable access and provide the necessary support for using the code upon reasonable request.
